# Relating factors to wearing personal radiation protectors among healthcare professionals

**DOI:** 10.1186/s40557-016-0144-x

**Published:** 2016-10-20

**Authors:** Yunjeong Heo, Hosun Chun, Seonghoon Kang, Wonjin Lee, Taewon Jang, Jongtae Park

**Affiliations:** 1 Department of Occupational and Environmental Medicine, Korea University Ansan Hospital, Ansan-si, Gyeonggi-do Republic of Korea; 2 Department of Preventive Medicine, Korea University College of Medicine, Seoul, Republic of Korea; 3 Department of Occupational and Environmental Medicine, Hanyang University Guri Hospital, Seoul, Republic of Korea

**Keywords:** Personal radiation protectors, Healthcare professionals, Preventive measures

## Abstract

**Background:**

With increasing use of medical radiologic procedures, wearing proper protector should be emphasized to reduce occupational radiation exposures. This research describes the rates of lead apron wearing for radiation protection and assessed occupational factors related to wearing rates for various types of healthcare professionals.

**Methods:**

We conducted a self-administered questionnaire survey through a website, on-site visits, fax, and mail. Of the 13,489 participants, 8858 workers who could not completely separate themselves from radiological procedure areas. Their general characteristics (sex and age), work history (job title, duration of employment, and hospital type), and practices (frequency of radiation procedures, ability to completely separate from radiation, and frequency of wearing protective lead aprons) were examined.

**Results:**

The mean rate of lead apron wearing during radiologic procedures was 48.0 %. The rate was different according to sex (male: 52.9 %, female: 39.6 %), hospital type (general hospital: 63.0 %, hospital: 51.3 %, clinic: 35.6 %, dental hospital/clinic: 13.3 %, public health center: 22.8 %), and job title (radiologic technologist: 50.3 %, doctor: 70.3 %, dentist/dental hygienist: 15.0 %, nurse/nursing assistant: 64.5 %) (*p* < 0.001). By logistic regression analysis stratified by job title, use of lead aprons by radiologic technologists and nurses/nursing assistants was associated with hospital type and exposure frequency score. For doctors, apron wearing was associated with employment duration. For dentists/dental hygienists, apron wearing was associated with the exposure frequency score.

**Conclusions:**

To improve working environments for healthcare professionals exposed to radiation, it is necessary to consider related factors, such as job title, duration of employment, and hospital type, when utilizing a planning and management system to prevent radiation-related health problems.

## Background

The use of ionizing radiation in hospitals is increasing worldwide, as more diagnostic x-ray, fluoroscopy, angiogram, dental x-ray, computed tomography (CT), nuclear medicine imaging, radiotherapy, and experimental procedures are performed, and radiation workers are increasing stepwise [1]. In 2014, there were 71,096 radiation workers in South Korea, which was 5.6 times more than the 12,652 workers in 1996. Among radiation workers, radiation technologists, physicians, and dentists account for 75 % of the total population, and dental hygienists, nurses, and nursing assistants account for the rest [2].

The International Commission on Radiological Protection (ICRP) provides international recommendations and guidance on all aspects of protection against ionizing radiation. In South Korea, the National Dose Registry follows the guidelines and monitors personal radiation exposure doses, which are not to exceed 50 mSv/year and 100 mSv/5 years, accordingly to the Regulations for Safety Management of Diagnostic Radiation. In 2014, 98.4 % of radiation workers monitored by the National Dose Registry of Korea were exposed to less than 5 mSv of diagnostic radiation, and 0.1 % of workers were exposed to more than 20 mSv. The mean of radiation exposure dose was 0.41 mSv, and it has decreased annually for the past five years [2].

Radiation exposure to members of the general population or other hospital workers almost always occurs within the low-dose range. Low-dose radiation means less than 100 mSv [3], and most international organization reports support a linear no-threshold model, in which there is no threshold for low-dose radiation that potentially causes cancer or genetic disorders [3–5].

Thus, it is necessary to reduce occupational radiation exposure as much as possible by employing prevention strategies, such as proper time, distance, and shielding techniques [6]. Among those strategies, shielding effectively reduces exposure, and wearing personal protective equipment to shield inevitable direct or indirect radiation exposure is advisable. Many studies suggest that it is necessary to use protective equipment when potentially being exposed to radiation. Simple protective equipment used by doctors and nurses conducting endoscopic retrograde cholangiopancreatography (ERCP) could reduce more than 90 % of radiation exposure doses [7]. Lead gloves reduce more than 99 % of radiation exposure doses during portable cervical spine radiography in the emergency room [8]. Lead aprons with thyroid collars reduce more than 97 % of radiation doses annually [9].

However, past studies have reported that radiation workers have insufficiently used the protective measures. There are 7.0–12.3 % of radiation technologists who did not use lead aprons [10–12]. Another study reported that no shielding screens or aprons were present in the emergency room during CT or simple radiography [13]. Some studies about radiation safety management have reported differences in knowledge, attitudes, and behavior between nurses and dental hygienists, and usually, the knowledge score was not equal to the behavior score [14–18].

Therefore, we conducted a study of radiation workers in South Korean hospitals to investigate radiation protection statuses for several occupations. The purpose of this paper is to describe and analyze the status of radiation protective behaviors and related occupational factors in order to provide baseline results for preventive management of healthcare professionals.

## Methods

### Subjects

Detailed information on the overall methodology was reported in a previous study [19]. Briefly, the target population for this study included all subjects registered with the National Dose Registry in 2011. National Dose Registry (NDR) is maintained for all radiation workers who use medical x-ray equipment installed in medical institutes. The NDR monitors dosing in these workers. Information regarding personal identification numbers, districts, job classification, and dose data are collected in the registry. Among the 15,501 subjects who responded to the survey, 2012 subjects (13.0 %) were excluded for missing values. From the remaining 13,489 subjects (87.0 %), 4631 subjects (29.9 %) responded that they spent time “separated from patients completely (e.g., control room or, console)”, and thus, they were also excluded. Finally, the remaining 8858 subjects (57.1 %) were analyzed.

### Study process

From April 2012 to May 2013, we conducted a self-administered questionnaire survey through a website, on-site visits, fax, and mail. We used a web-based system (http://www.rhs.kr/method/question_agree.asp) for the website survey. We visited occupational continuing education courses and conferences for radiologic technologists to administer the on-site surveys. We also sent questionnaires via mail, e-mail, and fax, based on National Dose Registry addresses. To solve the potential issue of selection bias in enrollment, we made an effort to expand recruitment and improve the participation rate. We contacted nonresponders and those from low participation areas or hospitals, and collected the questionnaire through telephone, mail, and fax in cooperation with public health centers. Two thousand six hundred twenty-six subjects were additionally recruited for an enrollment rate of 63 %.

### Questionnaire

The questionnaire included demographics (sex and age), work history (job title, duration of employment, and hospital type), and work practices (frequency of radiation exposure, frequency of separation from radiation during procedures using a 4-tiered percentage scale [0 %, <25 %, 25–75 %, and >75 %], and frequency of wearing protective lead apron). Job titles were categorized into four homogenous exposure groups: radiologic technologist, doctor, dentist/dental hygienist, and nurse/nursing assistant. Weekly radiation procedure frequency was verified for each routine diagnostic X-ray, mammography, panoramic radiography, cephalometric radiography, portable X-ray, CT, C-arm, fluoroscopy, interventional radiography, radiotherapy, and nuclear medicine imaging procedure using the Likert Scale, considering each procedure’s radiation dose per time. Exposure frequency score is a variable used in our study to evaluate the accumulated weekly radiation procedure frequency. It is calculated from the weekly radiation procedure frequency that we collected from the questionnaires. As workers were exposed to several different procedures each week, we added each Likert Scale value and developed the exposure frequency score. It represents a cumulative sum of the frequencies of each procedure performed in a week, and it increase as more radiological procedures were done, regardless of the type of the procedures. The degree of protective lead apron use was classified to four groups (0 %, less than 25 %, 25–75 %, or more than 75 %). We tentatively defined the wearing group as workers who use lead aprons at least 25 % of the time.

### Data analysis

The characteristics of study subjects were presented using descriptive statistics. To compare the lead apron wearing rate, the chi-squared test was used. We stratified the subjects by job titles, while considering various occupational factor distributions depending on the job. Using stratified data, we also conducted logistic regression of lead apron use with age, sex, job title, duration of employment, hospital type as independent variables. Age, duration of employment, and exposure frequency scores were processed as continuous variables. All analyses were performed in SPSS version 21.0 (SPSS, Chicago, IL, USA).

## Results

### General characteristics

Of the 8858 subjects who could not work completely away from the radioactive field, 5607 (63.3 %) were male, and 3251 (36.7 %) were female. Subjects 30–39 years old comprised the majority of the study population (3623; 40.9 %), followed by those 20–29 years old (2733; 30.9 %), 40–49 years old (1936; 21.9 %), and ≥50 years old (566; 6.4 %). Most subjects had worked less than 10 years (5523; 62.4 %), followed by those who worked between 10 and 20 years (2212; 25.0 %) and over 20 years (1123; 12.7 %). These subjects were most frequently located in a general hospital (3791; 42.8 %), hospital (1928; 21.8 %), clinic (1918; 21.7 %) dental hospital/clinic (874; 9.9 %), or public health center (347; 3.9 %). General hospitals have more than 100 beds, which distinguish them from regular hospitals for the purpose of this study. Subjects were most commonly radiologic technologists (7198; 81.3 %), dentist/dental hygienists (917; 10.4 %), nurses/nursing assistants (423; 4.8 %), and doctors (320; 3.6 %) (Table 1).

**Table 1:** General characteristics of study subjects

Characteristics	Number	Percent
Sex		
Male	5607	63.3
Female	3251	36.7
Age (years)		
20–29	2733	30.9
30–39	3623	40.9
40–49	1936	21.9
≥ 50	566	6.4
Duration of employment (years)		
< 10	5523	62.4
10–19	2212	25.0
≥ 20	1123	12.7
Hospital type		
General hospital^a^	3791	42.8
Hospital	1928	21.8
Clinic	1918	21.7
Dental hospital/clinic	874	9.9
Public health center	347	3.9
Job title		
Radiologic technologist	7198	81.3
Doctor	320	3.6
Dentist/dental hygienist	917	10.4
Nurse/nursing assistant	423	4.8

Equation 1 ^a^Hospital having 100 or more beds, including university hospitals

### Exposure frequency score and lead apron wearing rate, according to general characteristics

The mean exposure frequency score for all subjects was 6.76 ± 4.51. The exposure frequency score of male subjects was 7.20 ± 4.69, which was significantly higher than that of female subjects (5.99 ± 4.06, *p* < 0.05). Subjects 20–29 years old had the highest exposure levels (7.75 ± 4.28), followed by those 30–39 years old (6.93 ± 4.68), 40–49 years old (5.68 ± 4.26), and ≥50 years old (4.53 ± 3.71). There were similar trends with duration of employment. Subjects who worked less than 10 years had the highest exposure scores (7.28 ± 4.54), followed by those who worked 10-20 years (6.09 ± 4.41) and those who worked over 20 years (5.50 ± 4.12). As for hospital type, the score for the general hospital group was 8.21 ± 4.88, the hospital group scored 7.07 ± 4.42, the clinic group scored 4.41 ± 2.93, the dental hospital/clinic group scored 5.56 ± 3.32, and the public health center scored 5.14 ± 3.92. Radiologic technologists scored the highest (7.27 ± 4.59), followed by dentist/dental hygienists (5.14 ± 3.10), nurses/nursing assistants (4.56 ± 3.76), and doctors (2.87 ± 2.95) (Table 2).

**Table 2:** Exposure frequency score and lead apron wearing rate, according to general characteristics

Characteristics	Exposure frequency score	Wearing lead apron n (%)	Chi-squared test
Sex			<0.001
Male	7.20 ± 4.69	2967 (52.9)	
Female	5.99 ± 4.06	1289 (39.6)	
Age (years)			0.066
20–29	7.75 ± 4.28	1260 (46.1)	
30–39	6.93 ± 4.68	1790 (49.4)	
40–49	5.68 ± 4.26	927 (47.9)	
≥ 50	4.53 ± 3.71	279 (49.3)	
Duration of employment (years)			0.206
< 10	7.28 ± 4.54	2687 (48.7)	
10–19	6.09 ± 4.41	1027 (46.4)	
≥ 20	5.50 ± 4.12	542 (48.3)	
Hospital type			<0.001
General hospital	8.21 ± 4.88	2388 (63.0)	
Hospital	7.07 ± 4.42	990 (51.3)	
Clinic	4.41 ± 2.93	683 (35.6)	
Dental hospital/clinic	5.56 ± 3.32	116 (13.3)	
Public health center	5.14 ± 3.92	79 (22.8)	
Job title			<0.001
Radiologic technologist	7.27 ± 4.59	3620 (50.3)	
Doctor	2.87 ± 2.95	225 (70.3)	
Dentist/dental hygienist	5.14 ± 3.10	138 (15.0)	
Nurse/nursing assistant	4.56 ± 3.76	273 (64.5)	

The lead apron wearing rate for all subjects was 48.0 %, and the rate was higher for males (2967; 52.9 %) than for females (1289; 39.6 %). There were no significant statistical differences based on age groups (20–29 years, 46.1 %; 30–39 years, 49.4 %; 40–49 years, 47.9 %; ≥50 years, 49.3 %) or duration of employment (<10 years, 48.7 %; 10–20 years, 46.4 %; ≥20 years, 48.3 %). As for hospital type, 2388 general hospital workers (63.0 %), 990 hospital workers (51.3 %), 683 clinic workers (35.6 %), 79 public health center workers (22.8 %), and 116 dental hospital/clinic workers (13.3 %) wore lead aprons. With respect to job titles, the rate of lead apron wearing was highest among doctors at 70.3 % (225 subjects), followed by 64.5 % (273 subjects) for nurses/nursing assistants, 50.3 % (3620 subjects) for radiologic technologists, and 15.0 % (138 subjects) for dentists/dental hygienists. The differences in wearing between job titles were statistically significant (*p* < 0.001) (Table 2).

### Factors associated with the lead apron wearing rate according to the job title

Logistic regression analysis was performed using dependent variables (sex, age, duration of employment, hospital type, and job title) stratified by job title groups, and the factors associated with the lead apron wearing rate were different according to job title. (Table 3)

**Table 3:** Factors associated with the lead apron wearing rate

	Adjusted OR^a^ (95 % CI)
Radiologic technologist	
Duration of employment (years)	1.009 (0.986–1.033)
Exposure frequency score	1.150 (1.125–1.176)
Hospital type	
General hospital	1.000
Hospital	0.956 (0.772–1.183)
Clinic	0.378 (0.314–0.455)
Dental hospital/clinic	0.045 (0.018–0.113)
Public health center	0.103 (0.077–0.137)
Doctor	
Duration of employment (years)	1.062 (1.016–1.110)
Exposure frequency score	1.507 (1.158–1.961)
Hospital type^b^	
General hospital	1.000
Hospital	1.364 (0.317–5.875)
Clinic	0.681 (0.254–1.826)
Dentist/dental hygienist	
Duration of employment (years)	0.978 (0.970–1.018)
Exposure frequency score	0.938 (0.888–0.991)
Hospital type^c^	
Dental hospital/clinic	1.000
Public health center	3.144 (1.808–5.469)
Nurse/nursing assistant	
Duration of employment (years)	0.949 (0.899–1.003)
Exposure frequency score	1.275 (1.162–1.400)
Hospital type^d^	
General hospital	1.000
Hospital	0.396 (0.168–0.933)
Clinic/Dental hospital, dental clinic/Public health center	0.178 (0.053–0.599)

^a^Adjusted for age, sex, duration of employment, exposure frequency score and hospital type

^b^Subjects of dental hospital/clinic and Public health center is absent

^c^Subjects of general hospital, hospital and clinic is absent

^d^Subjects excepting general hospital and hospital are few

For radiologic technologists, the odds ratio [OR] (95 % confidence interval [CI]) related to exposure frequency score were 1.150 (1.125–1.176), which is a significant association with lead apron wearing. For hospital type, the ORs (95 % CI) were 0.378 (0.314–0.455) for the clinic, 0.103 (0.077–0.137) for the public health center, and 0.045 (0.018–0.113) for the dental hospital/clinic, compared to general hospitals.

For doctors, the ORs (95 % CI) for duration of employment and exposure frequency score were 1.062 (1.016–1.110) and 1.507 (1.158–1.961), which were statistically significant. Hospital type was not significantly associated with lead apron wearing (OR, 1.364; 95 % CI, 0.317–5.875).

For dentists/dental hygienists, the OR (95 % CI) for exposure frequency score was 0.938 (0.888–0.991), which was statistically significant. Regarding hospital type, the OR (95 % CI) for public health centers was 3.144 (1.808–5.469), which was statistically significant. Duration of employment was not significantly associated with lead apron wearing.

For nurses/nursing assistants, the OR (95 % CI) for exposure frequency score was 1.275 (1.162–1.400). Regarding hospital type, the OR of hospitals was 0.396 (0.168–0.933), and the OR for clinics/dental hospitals/clinics/public health centers was 0.178 (0.053–0.599), which were statistically significant. The duration of employment was not significantly associated with lead apron wearing.

## Discussion

In this study, factors related to lead apron wearing were different based on job titles. The apron-wearing rate for dentists/dental hygienists decreased with increasing exposure frequency scores, contrary to radiologic technologists and nurses/nursing assistants. In addition, the rates were decreased in small hospitals for all job types, except for doctors associated with short employment duration.

According to job titles, the rate difference was definite, with 70.3 % of doctors and 15.0 % of dentists/dental hygienists wearing lead aprons. There are few studies about the rate for only radiologic technologists, and the rates ranged from 83.1–93.0 % in all settings and 83.7–86.4 % for those in clinics [10–12]. Although rate itself was considerably low due to use of different apron wearing scales, declining apron wearing rates tended to be seen at smaller hospitals as in this study [17, 20, 21]. This suggests radiation protection management should focus on medium-to-small hospitals.

Our findings of a significant, positive association between employment period and the use of lead apron is in line with the results of other studies on radiation safety management for radiologic technologists, nurses, and dental hygienists. In these studies, the behavior scores about radiation protection, including wearing lead aprons, were low in subjects with short employment periods [17, 22–24], and the knowledge of radiation safety management correlated with attitudes and behavior about radiation safety management. Thus, it is important to educate subjects with short careers about the health risks of radiation and on how to protect themselves from it in a detailed and specific manner to enhance protective behaviors, such as lead apron wearing

The lead apron wearing rate and the associated exposure frequency of dentist/dental hygienists was different from that of participants with other job titles, as we have previously mentioned. Although the radiation dose per dental procedure is quite low in comparison with other procedures, repetitive low-dose exposure risk exists because of the increasing number of orthodontic, prosthodontic, and implant procedures [18]. Most dental hospital/clinics have more than one radiographic device [25], but the radiation safety management performance scores were relatively low [18, 26]. The study participants from dental facilities also had the lowest rate of lead apron wearing in the study. Considering the significant negative association between exposure frequency and the wearing of lead aprons in these technicians, appropriate educational efforts should be made to improve the use of protective garments in this group.

This study had some limitations. First, it is difficult to figure out the temporal relationship between lead apron wearing and associated factors, as this is cross-sectional design to reveal actual conditions. Secondly, this study subject does not represent the actual distribution of healthcare professionals due to the large proportion of radiologic technologists and relative absence of dentists/dental hygienists at general hospitals/hospitals. Finally, the lead apron wearing rate could be overestimated by defining the wearing group as workers who use lead aprons at least 25 % of the time. Nevertheless, this study was a meaningful to access the use of radiation protector including various types of healthcare professionals, and it could be used to administrate the preventive measures to reduce the radiation hazards in hospitals.

## Conclusions

This study was conducted to investigate the lead apron wearing rate of healthcare professionals and to analyze related factors that could be used to suggest radiation protection measures. The lead apron wearing rate was higher in doctors, nurses/nursing assistants, radiologic technologists, and dentists/dental hygienists, in that order. The lead apron wearing rate was mostly affected by hospital type, exposure frequency score and employment duration. Based on these results, the preventive measures should be specified by hospital type, job title and duration of employment to reduce radiation exposure. Additionally, the recognition of radiation hazards and continuing education is important to enhance the use of radiation protector.

## Abbreviations

CI: Confidence interval
CT: Computed tomography
OR: Odds ratio
